# Excision of a gastrointestinal stromal tumour in a dog using short‐wave infrared fluorescence imaging and indocyanine green

**DOI:** 10.1002/vms3.1506

**Published:** 2024-06-10

**Authors:** Jinyoung Choi, Sungin Lee

**Affiliations:** ^1^ Department of Veterinary Surgery, College of Veterinary Medicine Chungbuk National University Cheongju Republic of Korea

**Keywords:** dog, gastrointestinal stromal tumour, image‐guided surgery, indocyanine green, surgical margin

## Abstract

A 7‐year‐old castrated male Golden Retriever weighing 36.8 kg presented to the Veterinary Teaching Hospital with vomiting, anorexia and depression. After blood tests, radiographic, ultrasound and computed tomography examinations, a 7.85 × 5.90 × 8.75 cm mass was identified in the caecum. To visualise the tumour margin and improve the accuracy of tumour resection, intraoperative short‐wave infrared imaging using indocyanine green was performed during surgery. An indocyanine green solution was injected intravenously as a bolus of 5 mg/kg 24 h before surgery. Tumour resection was performed with a 0.5 cm margin from the fluorescent‐marked tissues. Histopathological examination revealed a diagnosis of a gastrointestinal stromal tumour (GIST) and the absence of neoplastic cells in the surgical margin, indicating a successful surgery. To our knowledge, this is the first case of a GIST resection in a dog using intraoperative short‐wave infrared imaging.

## Graphical abstract

The manuscript describes a clinical case of surgical resection of gastrointestinal stromal tumour using short‐wave infrared (SWIR) fluorescence imaging with indocyanine green in a dog. Using the method, the tumour location was identified and the resection margin was defined before proceeding with the surgery and histopathological examination confirmed complete excision of tumour. As in human medicine, the use of the SWIR method in gastrointestinal surgery for tumour resection was confirmed to be useful in veterinary medicine as well through this clinical case.

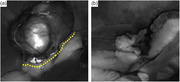

## 1 INTRODUCTION

Gastrointestinal tumours, such as leiomyosarcomas and gastrointestinal stromal tumours (GISTs), are uncommon mesenchymal neoplasms, representing approximately 3% of all tumours in dogs (Frgelecová et al., 2014; Russell et al., 2007). GISTs are derived from the interstitial cells of Cajal (Montañés et al., 2019), which are pacemaker cells within the smooth muscle and neural plexus that control peristalsis and intestinal mortality (Gillespie et al., 2011; Russell et al., 2007). Previously, GISTs were considered similar to and categorised in the same class of gastrointestinal tumours as leiomyosarcomas; however, they have been differentiated from leiomyosarcomas by advances in immunohistochemical staining techniques (Miettinen & Lasota, 2001; Nishida & Hirota, 2000). Adjuvant therapies such as radiation and chemotherapy are ineffective for the treatment of GISTs; therefore, surgical resection is always the main treatment method (Parab et al., 2019).

Several surgical techniques and procedures have been developed over the last few decades to enhance surgical outcomes, such as fluorescence imaging using indocyanine green (ICG; Alander et al., 2012). After intravenous (IV) injection of ICG, it binds to plasma proteins, with ICG–plasma proteins complexes expressing light at a wavelength of approximately 805–830 nm (Holt et al., 2014; Vahrmeijer et al., 2013). Owing to the enhanced permeability and retention effect in tumours, ICG complexes accumulate in tumour cells (Frost et al., 2003; Newton et al., 2020); tumours and tumour margins can be visualised using short‐wave infrared (SWIR) imaging devices (Holt et al., 2014).

Few veterinary cases of tumour resection using intraoperative SWIR imaging have been reported. The following case is the first case of intestinal tumour resection in a dog that underwent intraoperative ICG SWIR imaging to complete excision.

## 2 CASE HISTORY

**FIGURE 1:**
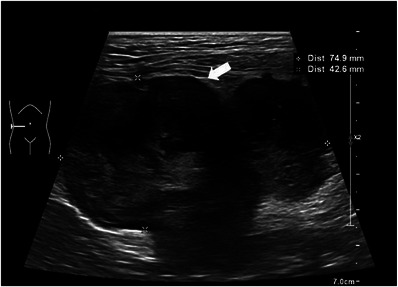
Ultrasonography of the mass (white arrow) in abdominal cavity. The mass has amorphous, irregular margins and hyperechoic peritoneum and mesentery are observed around the mass.

**FIGURE 2:**
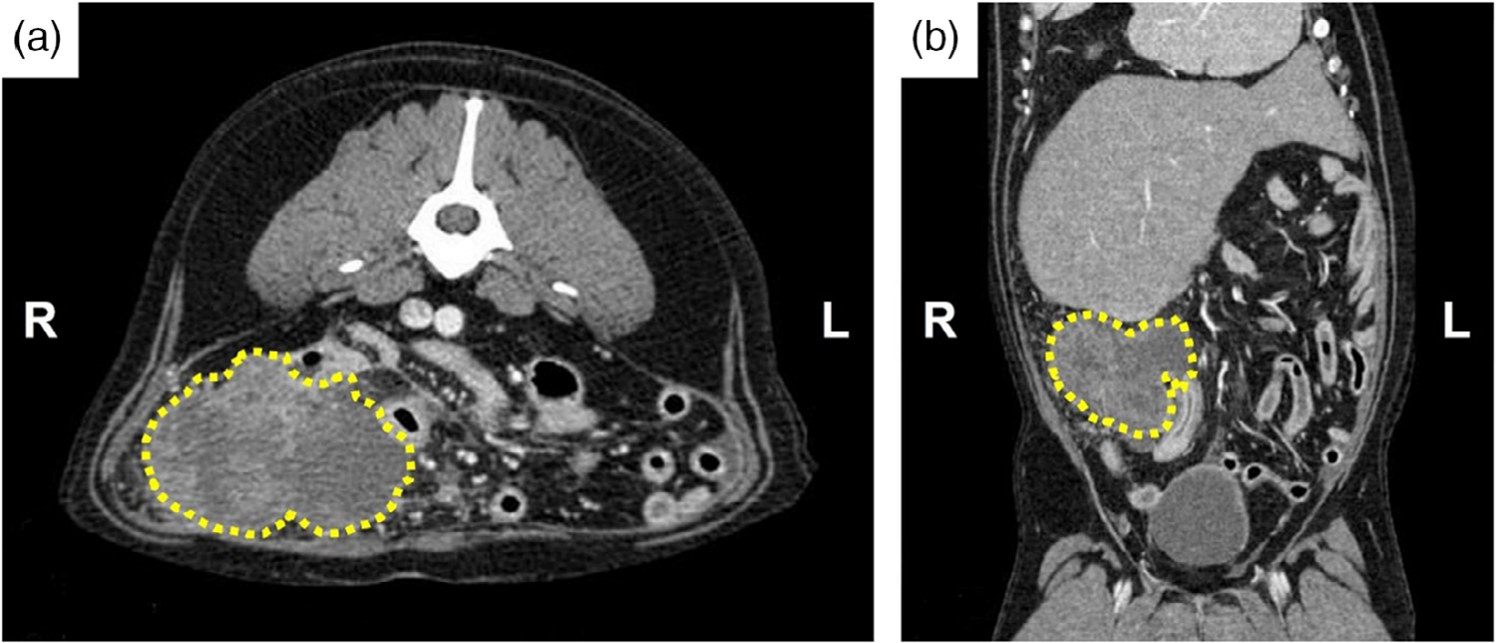
Pre‐surgical transverse (a) and coronal (b) computed tomography images of the patient. The mass (yellow dotted line) is located at the right caudo‐ventral region of caecum. The size of the mass is 7.85 × 5.90 × 8.75 cm (width × length × depth).

A 7‐year‐old castrated male Golden Retriever weighing 36.8 kg was referred to the Veterinary Teaching Hospital for vomiting, anorexia and depression. The patient underwent physical examination and general blood screening tests, identifying increased alkaline phosphatase (662 IU/L; reference interval, 29–97). Abdominal radiographs showed displacement and compression of the intestine by a mass in the abdominal cavity. Ultrasonography revealed an amorphous irregular marginal mass in the right middle abdominal cavity (Figure 1). Computed tomography revealed a 7.85 × 5.90 × 8.75 cm (width × length × depth) mass originating from the right caudo‐ventral region of caecum in transverse view (Figure 2a) and coronal view (Figure 2b). Ultrasonography‐guided fine‐needle aspiration cytology of the mass revealed a mesenchymal cell‐origin tumour.

Exploratory laparotomy was proposed to remove the abdominal mass identified by computed tomography. To identify the tumour and its margins, intraoperative ICG SWIR imaging was performed using SWIR fluorescence imaging devices developed by MetabpleBIO Inc. ICG (25 mg; Cellbiongreen, Cellbion Co.) was dissolved in 5 mL of enclosed sterile water, corresponding to a 5 mg/mL ICG solution. The solution was injected IV as a bolus of 5 mg/kg, totalling 36.8 mL, 24 h before surgery. The dog was hospitalised for 1 day for monitoring of the vital signs after ICG injection. During the hospitalisation, heart rate, respiratory rate, blood pressure and body temperature were monitored regularly, with no remarkable abnormalities noted.

**FIGURE 3:**
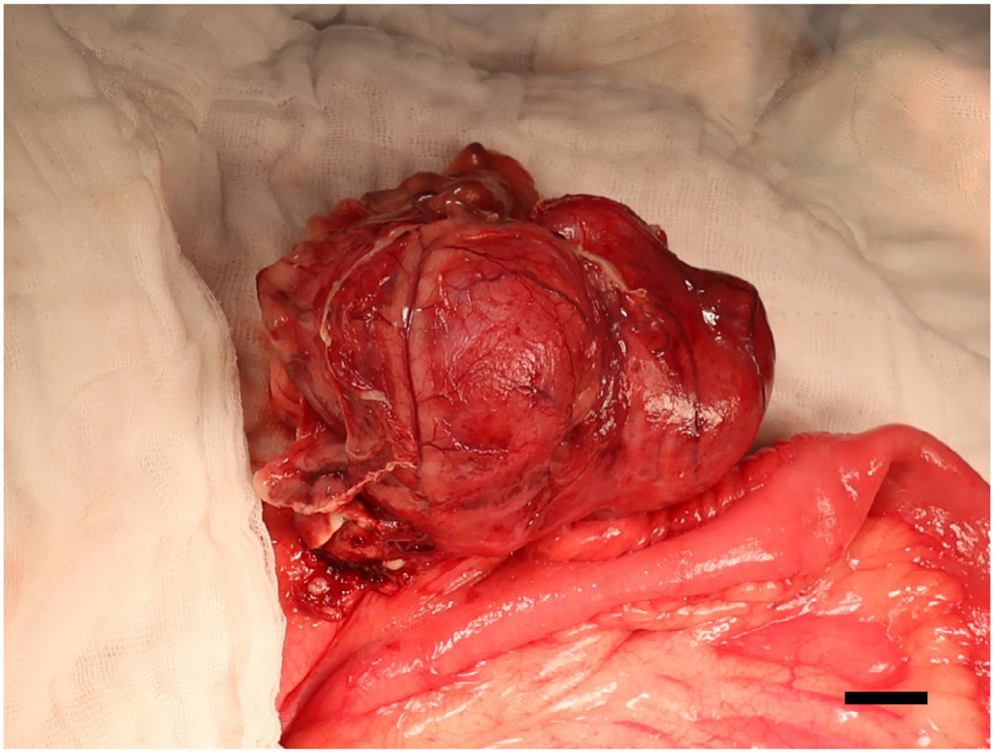
Intraoperative view of the caecal mass, with severe adhesion of the mesentery and intestine (scale bar, 1 cm).

**FIGURE 4:**
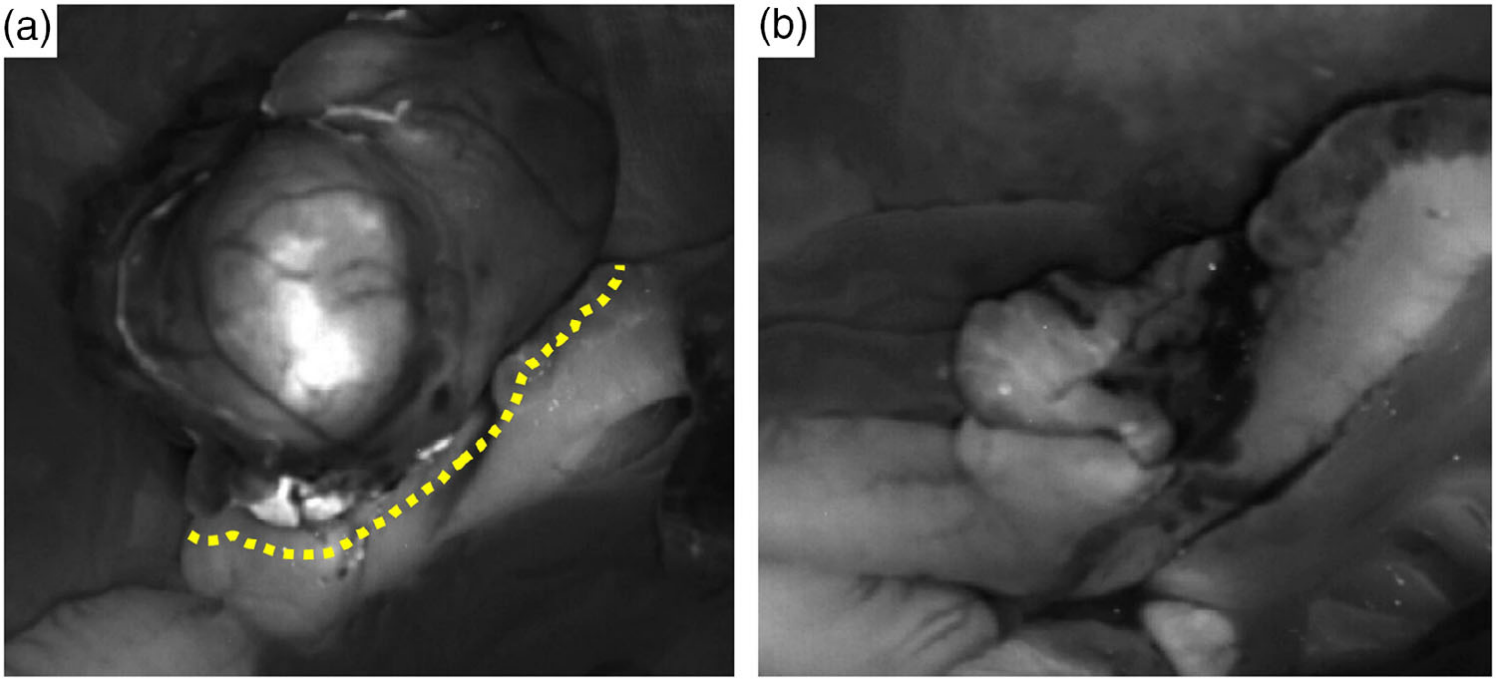
(a) Visualisation of the mass on the short‐wave infrared (SWIR) fluorescence imaging devices before resection. Note the fluorescence imaging of the margin of the mass and resection line (yellow dotted line). (b) After surgical resection, no residual fluorescent tissues were observed.

The patient was premedicated IV with cefazolin at a dose of 22 mg/kg (Cephazolin sodium, Chong Kun Dang Pharmaceutical Corporation), famotidine 1 mg/kg (Doga gaster, Donga‐ST) and midazolam 0.2 mg/kg (Midazolam, Bukwang Pharmaceutical Corporation). General anaesthesia was induced with 6 mg/kg of IV propofol (Provive, 1%, Myungmoon Pharm) and maintained with isoflurane (Terrell, Piramal Critical Care) after intubation using an 8.5 mm endotracheal tube. The dog was positioned in dorsal recumbency, and a midline incision using a No. 15 blade was made to access the caecum. The mass was confirmed to be derived from the caecum, with severe adhesions observed in the mesenteries and intestines (Figure 3). After removing the mass from the abdominal cavity, intraoperative SWIR imaging was performed, visualising the fluorescence of the ICG–protein complexes that had infrared contrast content with a peak emission of 808 nm (Figure 4a). Visually, fluorescence was observed approximately 2–3 mm away from site of the tumour, although the exact distance could not be measured due to technical limitations. Doyen forceps were applied 0.5 cm away from the end region of fluorescence imaging at the ileocaecal–colic junction. The resection was performed at the site where the mass was located in the caecum and the mass was excised parallel to the intestine rather than resecting the intestinal segment and anastomosis. After mass resection, the residual fluorescent tissues were assessed at the surgical sites using SWIR fluorescence imaging (Figure 4b). After confirming the absence of fluorescent regions, the resection region was sutured in a simple interrupted pattern using PDS II (Polydioxanone suture II 4‐0, Ethicon). After abdominal lavage, an active suction device was placed in the abdominal cavity, and the operation was completed with the closure of the body wall, subcutaneous tissue and skin.

**FIGURE 5:**
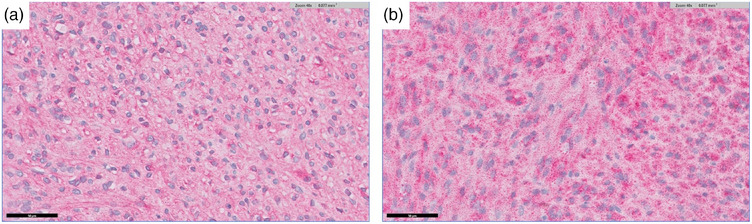
Histopathology and immunohistochemical staining of the mass are compatible with a diagnosis of malignant gastrointestinal stromal tumour. Images show neoplastic spindle cells exhibiting positive immunoreactivity for (a) C‐kit and (b) C‐kit, DOG1 immunohistochemistry (×40, scale bar 50 µm).

The removed mass underwent histopathological examination (IDEXX Laboratories) to identify its origin and histopathology. A tissue‐marking dye (Davidson Marking System, Bradley Products) was applied to the resected section of the intestinal margin to evaluate the surgical margin. The pathological examination revealed a spindle cell sarcoma, requiring a differential diagnosis between GIST and leiomyosarcomas using immunohistochemical staining. Histopathological and immunohistochemical findings, which were positive for C‐kit (Figure 5a) and DOG1 (Figure 5b), supported the diagnosis of malignant GIST. Neoplastic cells were absent in the dye‐marked areas. The mitotic counts were 3 per 10 high‐power fields, and lymphovascular invasion was not observed. Additionally, the intestinal section margin was complete without neoplastic cells.

The patient was discharged 3 days after surgery with prescription of antibiotics, and postoperative analgesia was managed with transdermal fentanyl patch at a dose of 25 µg/h (Durogesic D‐Trans patch 25 µg/h, Janssen Korea). After the surgical excision, the dog did not receive adjuvant systemic chemotherapy because of its low therapeutic effect and high treatment costs. The patient regularly presented for evaluation of local recurrence and metastasis through thoracic and abdominal radiographs as well as abdominal ultrasonography. The examination intervals were scheduled for 1 month, 3 months after surgery. And thereafter, follow‐up presentations were conducted every 3 months. Until the last presentation, there were no evidences of metastasis and recurrence detected in the examinations.

## 3 DISCUSSION

In humans, GISTs most commonly arise in the stomach, followed by the small intestine and colon (Miettinen & Lasota, 2001). In contrast, in dogs, GISTs occur most commonly in the caecum (Frost et al., 2003; Gillespie et al., 2011; Miettinen & Lasota, 2001), which is where the tumour was located in this case. Leiomyosarcomas appear similar to GISTs; these mesenchymal tumours were reclassified as expressing CD117 (KIT; Gillespie et al., 2011; Russell et al., 2007). Dogs with GIST are likely to be older at presentation than those with leiomyosarcoma; therefore, dogs with GIST might present higher perioperative mortality because of caecal perforation and septic peritonitis than others (Maas et al., 2007). Complete intestinal resection and anastomosis are the main surgical treatments for intestinal neoplasia including GIST (Tobias, Karen M., & Johnston, S. A., 2017). Generally, at intestinal tumour surgery, it is advised to perform resection with approximately 3 cm margin of normal intestinal tissue of the cranial and caudal directions from the tumour to ensure adequate surgical margin (Tobias, Karen M., & Johnston, S. A., 2017). The recurrence rates of GIST are approximately 7%–29% in several studies (Del Alcazar et al., 2021; Maas et al., 2007). Therefore, performing surgical margin to completely excise tumours is important during surgery.

To identify the tumour's origin and adequate surgical margins for minimal resection, intraoperative SWIR fluorescence imaging was performed in this case. In standard surgical methods, gastrointestinal tumours, with the affected bowel, should be resected 3 cm from the grossly normal bowel in each direction to prevent recurrence and residual neoplastic tissues (Tobias, Karen M., & Johnston, S. A., 2017). However, using SWIR fluorescence imaging, resection was performed at a surgical margin 0.5 cm apart from the fluorescent marker. During surgery, it allowed for the real‐time visualisation of tumours and non‐visible tumour and more accurate planning of surgical margins. The method can improve the accuracy of tumour resection and reduce the risk of recurrence. Histopathological examination confirmed that the resected tumour was a GIST, and the absence of neoplastic cells in the surgical margin of the resection indicated a successful surgery.

To ensure adequate surgical margin, SWIR imaging has been applied in human medicine (Kalisvaart et al., 2022). Not only in human medicine, SWIR imaging is also used in veterinary medicine, to ensure the complete removal of tumours (Kim and Lee, 2022). It may improve metastases detection and reduce the recurrence risk (Sposito et al., 2022). Additionally, it can be used to visualise blood vessels and other structures during surgery, which can reduce the risk of iatrogenic injury (Slakter et al., 1995).

However, there are some limitations to its use in veterinary medicine. One is the need for specialised training in the equipment's use to visualise ICG, which may limit its use to experienced surgeons. To date, no studies have been conducted on the appropriate dosage and injection time of ICG for gastrointestinal surgery in dogs. In addition, ICG accumulates not only in tumours, but also in inflammatory cells (Alander et al., 2012). Finally, confirming that the use of the SWIR imaging during surgery is successful to achieve complete tumour resection is not possible based on the results of this single case.

In conclusion, SWIR imaging using ICG is a promising technique for improving the complete surgical margins in dogs and cats with gastrointestinal tumours. To our knowledge, this is the first reported case of a gastrointestinal tumour resection using SWIR imaging. Further research is needed to evaluate the long‐term outcomes of this technique and to identify ways to improve its accessibility and usability in veterinary clinical practice and its adverse effects.

## AUTHOR CONTRIBUTIONS

**Jinyoung Choi**: Conceptualisation (equal); data curation (equal); investigation (lead); methodology (equal); validation (equal); visualisation (equal); writing – original draft (lead); writing – review and editing (equal). **Sungin Lee**: Data curation (equal); formal analysis (equal); investigation (supporting); methodology (equal); supervision (lead); validation (equal); visualisation (equal); writing – original draft (supporting); writing – review and editing (equal).

### ETHICS STATEMENT

Not applicable.

### CONSENT FOR PUBLICATION

Written informed consent for publication of the clinical details was obtained from the dog's owner.

### CONFLICTS OF INTEREST STATEMENT

The authors declare no conflicts of interest.

### PEER REVIEW

The peer review history for this article is available at https://publons.com/publon/10.1002/vms3.1506.

## Data Availability

Data sharing not applicable to this case report as no datasets were generated or analysed during the current report.
